# Factors influencing SARS-CoV-2 transmission and outbreak control measures in densely populated settings

**DOI:** 10.1038/s41598-021-94463-3

**Published:** 2021-07-27

**Authors:** Rachael Pung, Bernard Lin, Sebastian Maurer-Stroh, Fernanda L. Sirota, Tze Minn Mak, Sophie Octavia, Junxiong Pang, Iain Beehuat Tan, Clive Tan, Biauw Chi Ong, Alex R. Cook, The Singapore COVID-19 Outbreak Research Team, Doreen Tan, Qin Xuan Chua, Samuel Zeng Rong Chong, Han Fang Koh, Elena Yap, Priscilla Sia, Ze Ren Tan, Fong Sin Lee, Jun Kang Enan Goh, Peou Socheata Monica Suor, Julian Xiao Li Ang, Vernon J. Lee

**Affiliations:** 1 Ministry of Health, Singapore, Singapore; 2 Ministry of Manpower, Singapore, Singapore; 3 Government Technology Agency, Singapore, Singapore; 4 Bioinformatics Institute, Agency for Science, Technology and Research, Singapore, Singapore; 5 Department of Biological Sciences, National University of Singapore, Singapore, Singapore; 6 National Public Health Laboratory, National Centre for Infectious Diseases, Singapore, Singapore; 7 Saw Swee Hock School of Public Health, National University of Singapore, Singapore, Singapore; 8 National University Health System, Singapore, Singapore; 9 Department of Medical Oncology, National Cancer Centre, Singhealth, Singapore, Singapore; 10 Genome Institute of Singapore, Agency for Science, Technology and Research, Singapore, Singapore; 11 Duke-NUS Medical School, Singapore, Singapore; 12 Singapore Armed Forces Headquarters Medical Corps, Singapore, Singapore; 13 Sengkang General Hospital, Singhealth, Singapore, Singapore; 14 Singhealth Duke-NUS Academic Medical Centre, Singapore, Singapore; 15 Ministry of Health Holdings Pte Ltd, Singapore, Singapore

**Keywords:** Viral infection, Epidemiology

## Abstract

Starting with a handful of SARS-CoV-2 infections in dormitory residents in late March 2020, rapid transmission in their dense living environments ensued and by October 2020, more than 50,000 acute infections were identified across various dormitories in Singapore. The aim of the study is to identify combination of factors facilitating SARS-CoV-2 transmission and the impact of control measures in a dormitory through extensive epidemiological, serological and phylogenetic investigations, supported by simulation models. Our findings showed that asymptomatic cases and symptomatic cases who did not seek medical attention were major drivers of the outbreak. Furthermore, each resident had about 30 close contacts and each infected resident spread to 4.4 (IQR 3.5–5.3) others at the start of the outbreak. The final attack rate of the current outbreak was 76.2% (IQR 70.6–98.0%) and could be reduced by further 10% under a modified dormitory housing condition. These findings are important when designing living environments in a post COVID-19 future to reduce disease spread and facilitate rapid implementation of outbreak control measures.

Over the course of the COVID-19 pandemic, control measures in various countries have shifted from broad interventions such as widespread lockdowns and closures, to risk-based and targeted strategies in particular sub-populations or environments to minimise disruption to social and economic activities^1–3^. Urban settings such as cities are vulnerable to COVID-19 spread as close-living environments (e.g. student or worker dormitories and hostels, senior living facilities, military camps, prison facilities) generally lead to higher degrees of social interactions which then contribute to communicable disease spread^4,5^.

In Singapore, local community clusters involving imported cases and the largely naive local population were identified in late March, 2020^6^. Some clusters occurred at construction worksites and commercial areas frequented by foreign workers. In the same period, a surge of COVID-19 cases was identified across multiple foreign worker dormitories—the place of residence for about a quarter of the 1.4 million foreign workers in Singapore^7,8^. These dormitories were built with features such as common recreational and shared facilities, such as gyms, outdoor game courts, minimarts, and cooking areas^9^. These facilities provide foreign workers with a good living environment and promote social interactions. However, with thousands of residents in each dormitory, the living density in a dormitory is about 4.5 square metre per resident (in-line with the International Labour Organization standards)^10,11^. Due to the dense contact networks in these closed living environments, infectious diseases introduced to a dormitory could result in high levels of transmission. Understanding the combination of factors that could result in the surge of COVID-19 cases in dormitories would shed light on transmission patterns, and guide future housing solutions and outbreak interventions in dormitories and other similar densely populated settings to curb communicable disease transmission.

In this study, we developed an individual-based model of a dormitory and incorporated epidemiological and serological investigations findings to identify combinations of parameters that reproduced similar outbreak trajectory in the dormitory. We studied the temporal extent of missed infections and evaluated the effectiveness of the outbreak control measures under the current dormitory outbreak and in alternative scenarios: (i) baseline scenario: only case isolation and quarantine of roommates were implemented; (ii) enhanced response and physical distancing scenario: ground teams were deployed to expedite case isolation and enforce physical distancing measures but no strengthening of measures was applied to reduce the probability of infections occurring outside a room in the lockdown phase of the outbreak; (iii) modified dormitory setting scenario: dormitory layout with reduced number of residents per room and, en suite bathroom, shower and cooking facilities to ensure that persons under quarantine did not leave their rooms and movement restrictions across different levels for the remaining residents when the dormitory was under lockdown. Details of the respective interventions in each scenario are described in the Supplementary Information.

## Results

From March 23 to June 20, 2020, 2787 COVID-19 all-male cases with a median age of 33 (IQR 28–38) were identified in a dormitory cluster; none had recent travel history 14 days prior to the onset of symptoms or notification date.

In the pre-lockdown phase (before April 6, 2020), 12 of the initial cases were tested based on the doctor’s clinical evaluation while one case was tested as part of pneumonia surveillance. An additional 68 cases were identified to fulfil the suspect case definition. Five of the cases were each identified to be associated with at least one other COVID-19 cluster occurring in three worksites and a commercial area frequented by foreign workers. Over the course of the outbreak, three dormitory operations personnel developed COVID-19.

### Model fitting

**Figure 1:**
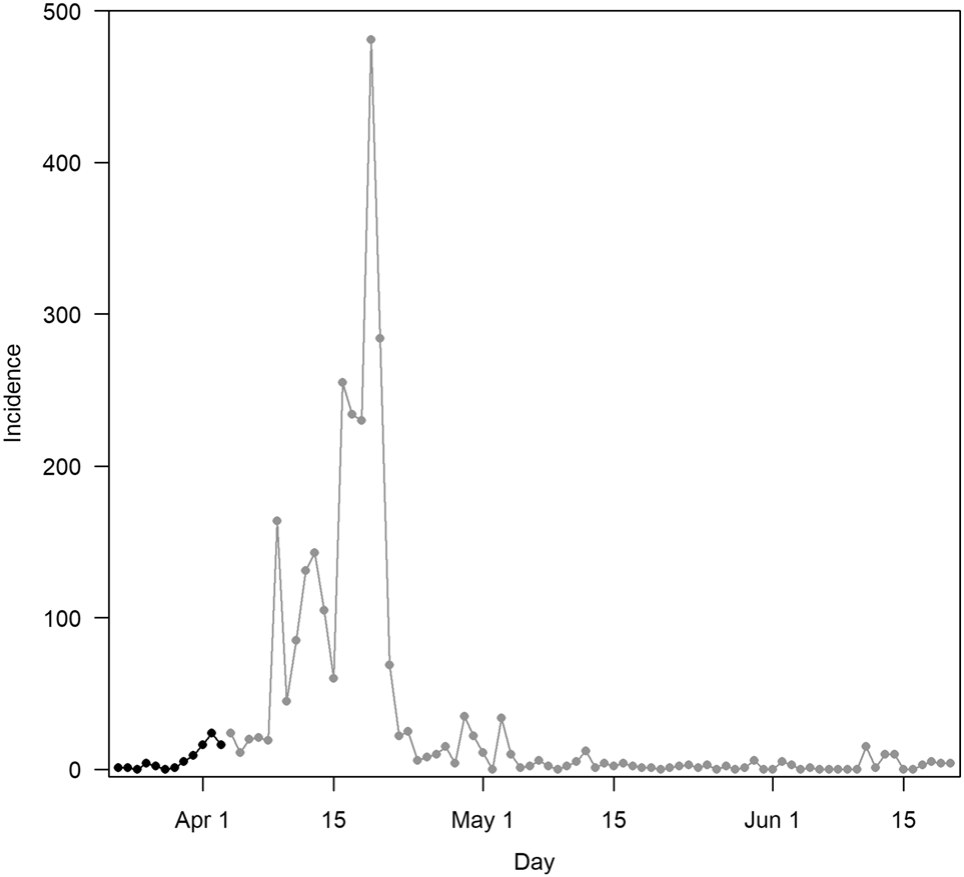
Outbreak trajectory in the dormitory. Observed symptomatic cases with onset on or before Apr 3, 2020(black dots, up to day 12 of the outbreak) were used for model fitting. Cases after Apr 3, 2020 (grey dots) were plotted based on notification dates if onset dates were not available and were not used for model fitting.

Following the lockdown, cases were observed to decline in mid-April 2020 (Fig. 1). The overall serology outcomes of the 7367 workers who participated in the seroprevalence survey showed that 72.0% (95% CI 70.9–73.0) of the workers were seropositive and these workers were not identified previously as having acute infection.

From the 10,000 sampled parameter combinations, we observed that about 13 cases (range 6–19) were required for a sustained outbreak to occur and 70.8% (95% CI 64.5–76.9%) of the infections were subclinical or asymptomatic cases with a relative infectiousness of 17.5% (95% CI 11.3–23.7%) that of a symptomatic case (Table 1). We estimated that about 43.4% (95% CI 32.7–54.2%) of the symptomatic cases sought medical attention and were tested before isolation. The probability of infection via contact with a susceptible roommate and others outside the room was 70.3% (95% CI 64.0–76.6%) and 54.9% (95% CI 41.9–67.9%) respectively before the strengthening of public health measures in the lockdown phase of the outbreak.

**Table 1:** Combination of factors that reproduced similar outbreak observations in the dormitory under study.

Parameter category	Parameter set	mean (95% confidence interval)
Disease transmission	Initial number of cases	13.9 (12.3–15.4)
	Proportion of asymptomatic cases (%)	70.8 (64.5–76.9)
	Relative infectiousness of an asymptomatic case (%)	17.5 (11.3–23.7)
	Probability of infection inside a room (%)	70.3 (64.0–76.7)
	Probability of infection outside a room (%)	54.9 (41.9–67.9)
Contact network	Mean number of random contacts form on the same level	9.9 (7.9–12.0)
	Mean number of random contacts form on different levels but same block	5.0 (3.9–6.1)
	Mean number of random contacts form in different blocks	2.6 (1.9–3.3)
Health seeking behaviour	Proportion of symptomatic cases seeking medical attention (%)	43.4 (32.7–54.2)
Effectiveness of public health measures	Probability that contacts with persons on the same level remains after social distancing (%)	38.4 (28.5–48.3)
	Probability that contacts with persons on different levels of the same block remains after social distancing (%)	42.3 (29.6–55.1)
	Probability that contacts with persons in different block remains after social distancing (%)	50.7 (38.0–63.4)
	Reduction in probability of infection outside a household (%)	21.6 (15.4–27.7)
	Days since deployment of ground officers when reduction in probability of infection outside a household occurred	3.9 (3.1–4.6)

For the outbreak to spread to other multiple blocks and levels of the dormitory by day 12, we estimated that the mean number of contacts formed by each individual (i) on the same level was 9.9 (95% CI 7.9–12.0), (ii) on the same block was 5.0 (95% CI 3.9–6.1), and (iii) in other blocks was 2.6 (95% CI 1.9–3.3).

### Outbreak intervention scenarios

**Figure 2:**
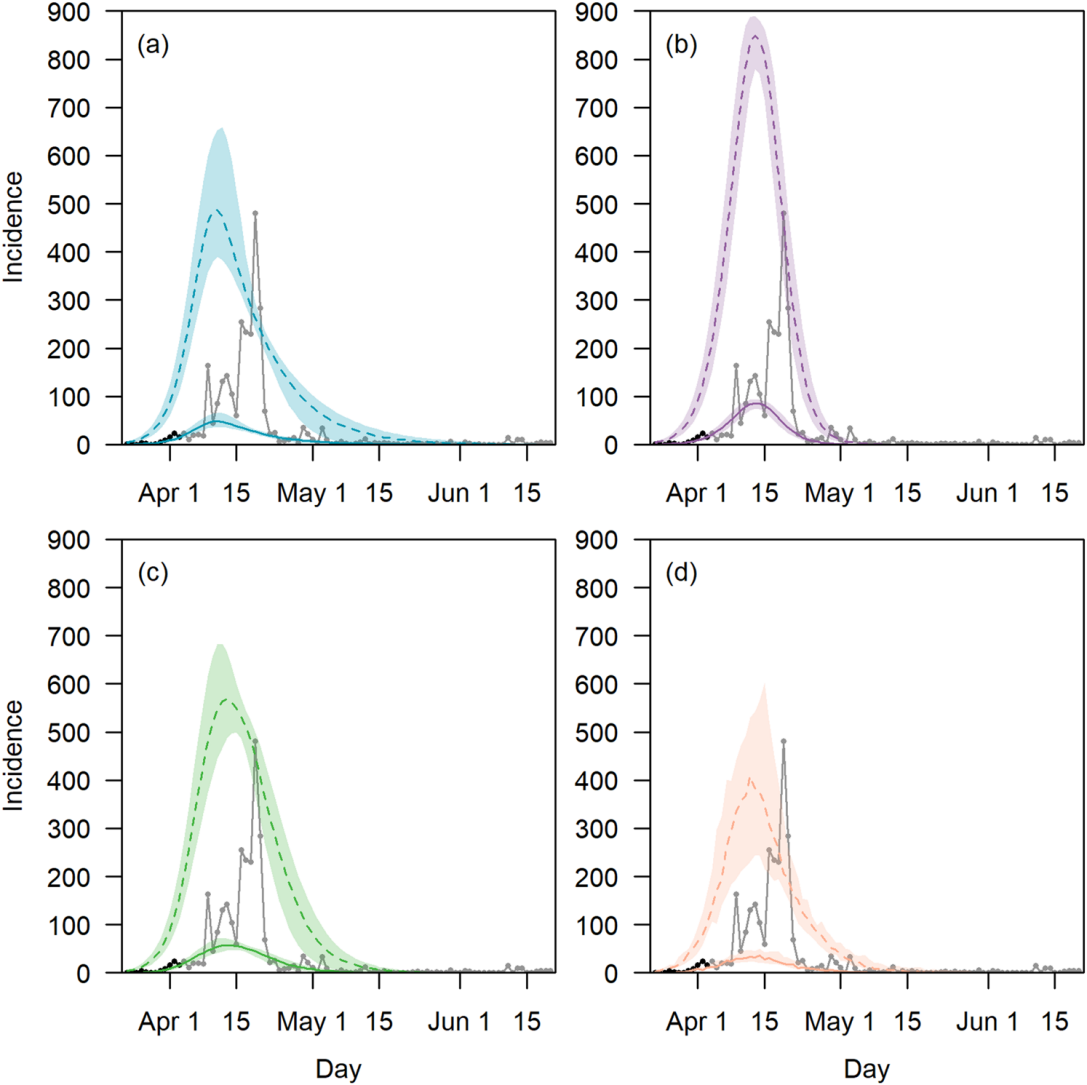
Outbreak trajectory in symptomatic cases who sought for medical treatment (solid line) and in all cases (dashed lines) with 95% confidence intervals (shaded area) by scenario (**a**) current outbreak scenario (**b**) baseline scenario, (**c**) enhanced response and social distancing scenario, (**d**) modified dormitory settings scenario. Observed symptomatic cases with onset on or before 3 Apr 2020 (black dots, up to day 12 of the outbreak) were used for model fitting. Cases after 3 Apr 2020 (grey dots and lines) were plotted based notification dates if onset dates were not available and were not used for model fitting due to incomplete information on onset dates for some of the symptomatic cases.

Based on the sampled parameter combinations, we estimated that the current outbreak peaked at 511 infections (IQR 407–732) on day 20 (IQR 19–21) with a final attack rate of 76.0% (IQR 70.6–98.0%) and the outbreak ending on day 92 (IQR 65–109) (Table 2 and Fig. 2a). We estimated that 87.3% (IQR 80.8–86.7%) of the infections were not detected over the course of the outbreak prior to the use of serology testing.

**Table 2:** Outbreak characteristics by intervention scenarios.

Outbreak outcomes	Current outbreak		Baseline			Enhance response and social distancing			Modified dormitory setting		
	Median	IQR	Median	IQR	p value	Median	IQR	p value	Median	IQR	p value
Final attack rate (%)	76.0	(70.6–98.0)	97.9	(97.7 –98.4)	10^–16^	93.1	(92.5–98.3)	10^–16^	66.4	(51.9–80.8)	10^–16^
Outbreak duration (days)	92	(65–109)	56	(52–60)	10^–16^	75	(63–83)	10^–16^	72	(59–100)	10^–16^
Peak outbreak size	511	(407–732)	905	(877–931)	10^–16^	602	(553–747)	10^–16^	415	(253–705)	10^–16^
Time to peak in outbreak (days)	20	(19–21)	22	(21–24)	10^–16^	23	(21–25)	10^–16^	22	(20–23)	10^–16^

The maximum daily number of infections was 905 (IQR 877–931) in the baseline scenario (Fig. 2b), 602 cases (IQR 553–747) in the enhanced response and physical distancing scenario (Fig. 2c). The time to outbreak peak in both scenarios remained largely unchanged relative to the current outbreak scenario. The overall attack rate was 97.9% (IQR 97.7–98.4%) with the outbreak ending within 56 days (IQR 52–60) in the baseline scenario (Fig. 2b). Under the modified dormitory setting scenario, the final attack rate was 66.4% (IQR 51.9–80.8%) (Fig. 2d).

**Figure 3:**
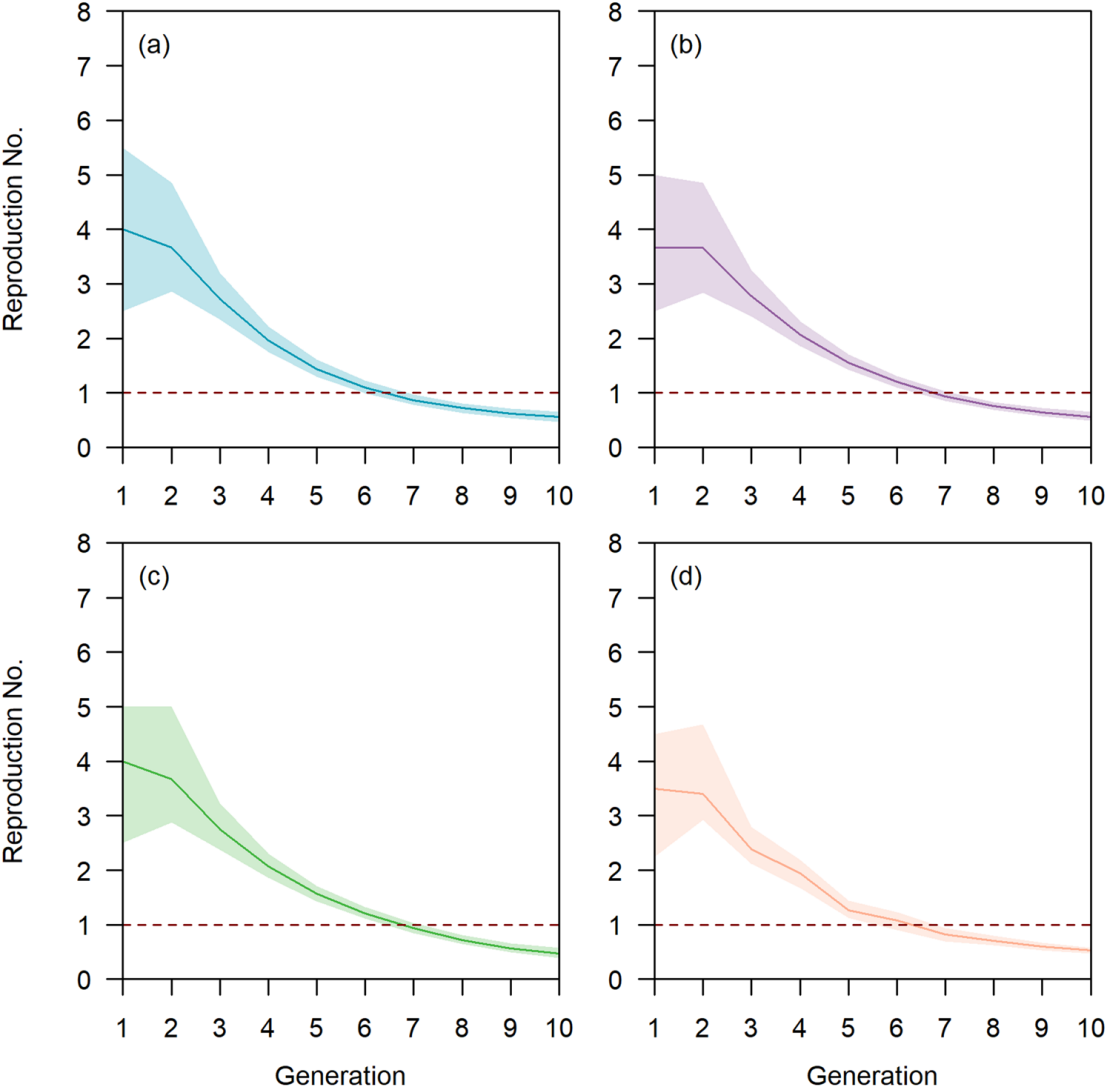
Reproduction number in symptomatic cases who sought for medical treatment (solid line) with 95% confidence intervals (shaded area) over generations by scenarios (**a**) current outbreak scenario (**b**) baseline scenario, (**c**) enhanced response and social distancing scenario, (**d**) modified dormitory settings scenario.

In the current outbreak scenario, the reproduction number declined from 4.0 (IQR 2.5–5.5) to 2.0 (IQR 1.8–2.2) by the fourth generation (Fig. 3a). In the baseline scenario, the reproduction number declined from 3.7 (IQR 2.5–5.0) to 2.0 (IQR 1.9–2.3) over 4 generations of transmission (Fig. 3b) and similarly from 4.0 (IQR 2.5–5.0) to 2.1 (IQR 1.9–2.3) in the enhanced response and social distancing scenario (Fig. 3c). Under the modified dormitory setting, this was reduced to 1.8 (95% CI 1.6–1.9) by the fourth generation (Fig. 3d). The initial reproduction number of 4.0 was at least five times the observed reproduction number of 0.8 among cases in the community over the same time period (unpublished data) (p value < 0.05). In all scenarios, the reproduction number fell below unity after seven generations of transmission.

### Phylogenetic outcomes

**Figure 4:**
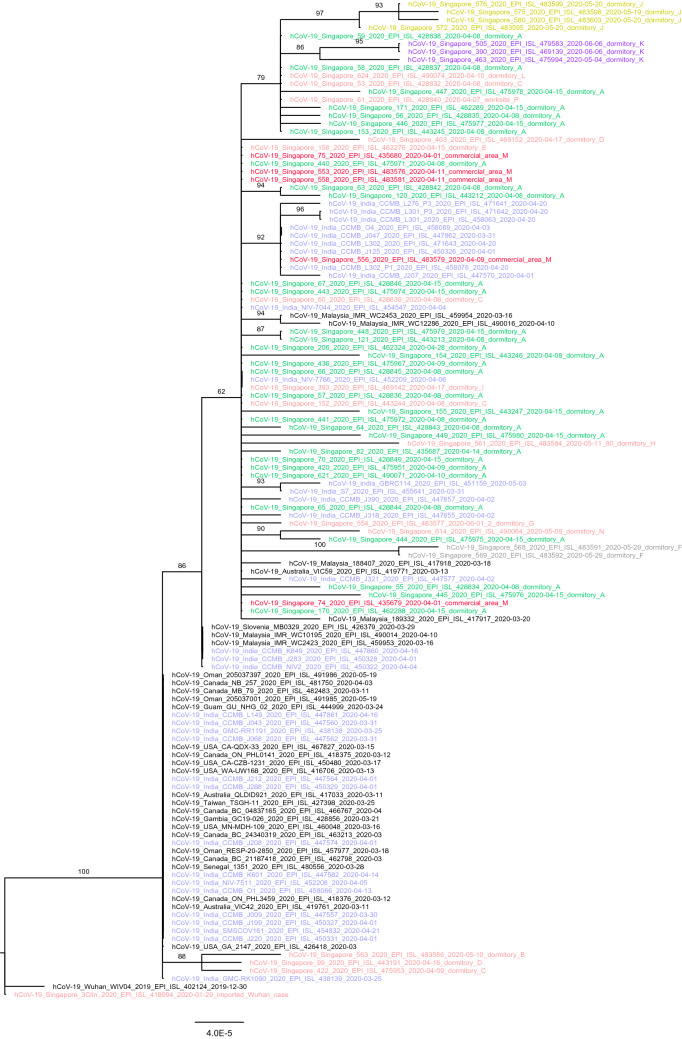
Phylogenetic tree plotted based on available samples from the dormitory cases and those linked to the dormitory outbreak. Selected genomes in the GISAID database was also plotted to place the sequences from locally notified cases into the global context.

Phylogenetic analyses showed that the genomes from the dormitory under study (dormitory A) were highly similar and belonged to the pangolin lineage B.6 (GISAID clade O). As shown in the phylogenetic tree, viruses belonging to this lineage circulated with high frequency within several countries in the region such as India, Malaysia and Australia and were different from those circulating in Wuhan, China (Fig. 4).

Locally, the sequences were highly similar to other genomes from a cluster associated with a popular commercial area (commercial area M) frequented by foreign workers and travellers (Fig. 4). Documented index cases of commercial M cluster preceded dormitory A outbreak. Furthermore, worksite P where workers from different dormitories were intermixing with workers from dormitory A had seeded the virus into dormitory L, K, J, C.

Distinguishable clusters characterized by unique genetic markers were found for 3 dormitories (dormitory J, K and F) (Fig. 4). Interestingly, while most samples from the commercial area M were found basal to the outbreak, there was one sample (Singapore/556) that was part of a separate cluster unique to other sequences from India, suggesting the possibility of multiple introductions via similar routes.

## Discussion

The spread of COVID-19 in densely populated residential areas have been documented in major cities and countries such as Australia^12^, South Korea^13^, India^14^ and Hong Kong^15^. In Singapore, epidemiological investigation of the initial cases in the dormitory under this study did not reveal a common source of infection. Some of the cases were associated with more than one COVID-19 clusters occurring at their worksites or social-recreational areas thereby increasing the chances of multiple introductions of COVID-19 infections into the dormitory from the foreign worker community. Furthermore, phylogenetic analysis of the genetic sequences showed close similarity with other circulating viruses in India and Malaysia, and hence, import-related transmission to the dormitory, directly or through other clusters, cannot be ruled out. Based on the epidemic trajectory, our modelling outputs suggest that there were approximately 13 cases that seeded the outbreak.

Given the uncertainties of the contact network patterns and disease transmission dynamics in a dormitory, we explored and showed that varied parameter combinations could result in similar observations in the early and late phases of the outbreak. As such, multiple interventions are essential to successfully contain an outbreak in these settings. In the current outbreak scenario, with strengthened outbreak containment measures, the overall attack rate was about 22% lower than the baseline scenario. The strengthened measures help to flatten the epidemic curve with peak cases more than 40% lower as compared to the baseline scenario. However, the outbreak duration in the current scenario was lengthened by about 1–2 months compared to the baseline. The surge in cases into the hundreds per day further justified the need for ground healthcare and operations teams to assist in managing ill residents in situ, as most of the infections were mild in this group of mostly young and healthy individuals. Despite the large number of dormitory cases, strict infection control observed by these workers ensured that infections in dormitory operations personnel and spill over into the community were kept to a minimum (in this dormitory, three ground officers were infected).

COVID-19 antibodies were detected in 72.0% of the 7367 persons with no prior PCR confirmation of SARS-CoV-2 infection. Our model estimated that about 71% of the infections in the current outbreak were attributed to subclinical asymptomatic infections. This estimate falls within the observed range of asymptomatic rates discussed in review studies and lies close to the upper limits^16,17^. We also estimated that 57% of the symptomatic cases did not seek medical attention and this could be attributed to mildly symptomatic persons who did not report symptoms. The overall extent of missed infections corroborates with a WHO report where 80% of the infections were mild or asymptomatic^18^. More could be done to educate individuals on the symptoms of COVID-19 and to encourage testing.

Our model estimated that each worker had approximately 18 contacts outside the household with nearly 80% of the contacts were formed within the same block and the remaining with persons in other blocks. The dense contact network facilitated disease transmission with the reproduction number in the dormitory exceeding 1 for six generations. This was several times higher than that observed in the community given the dense living conditions and because dormitories are specifically set up for social interactions among foreign workers. Hence, modified living environments for dormitories and similar living settings should be explored for the post-COVID-19 era to include increased living space, lowered occupancy per room and en suite facilities where possible^10^. The modelled number of infections under a modified dormitory setting would be reduced by at least 10%, and more if we incorporate a reduced probability of infection within the room given the increase in living area per individual. Furthermore, our model suggested that the majority of the random contacts outside the room were made with persons within the same block for a dormitory with shared facilities. Given that these persons could be exposed prior to the isolation of their infector, future outbreak containment could consider the movement restrictions of all persons in a block when a case of COVID-19 is identified. This would be at odds with the design of such living environments to promote social interaction but is necessary in the post-COVID-19 era to reduce the spread of infectious diseases in a naïve population. As the modification of the built environment of these residential areas comes at higher capital and operational cost, cost-effectiveness studies will need to be performed to determine the optimal build strategy.

Our study has some limitations. While there was regular disinfection and housekeeping in the dormitories, environmental transmission^19^ could have occurred but were not modelled. Cases in the lockdown phase of the dormitory outbreak were not used for model fitting as mass testing was conducted among dormitory residents to identify asymptomatic and symptomatic infections but the symptom onset dates were not routinely collected for symptomatic cases. The incomplete symptom onset dates for these cases resulted in reduced data available for model fitting. This potentially led to higher uncertainty in the daily incidence during the lockdown phase of the modelled current outbreak scenario as compared to the start of the outbreak (Fig. 2a). In the transmission model, we assumed that symptomatic cases who sought medical attention will do so upon onset. This is pertinent to the lockdown phase of the outbreak with increased residents’ awareness of the situation and delayed testing would reduce the effectiveness of the outbreak control measures. We also assumed that pre-symptomatic transmission could occur three days prior to symptoms onset and a case is infectious up to 14 days after symptoms onset. While these assumptions were made with reference to multiple studies^20–22^, there are numerous uncertainties and confounding factors influencing the exact profile of a case infectiousness since time of infection. Further studies on the infectious profile specific to workers in dormitories would help to refine the modelling assumptions. Also, we did not stratify the model by age and COVID-19 infection in older individuals could result in increased severity of symptoms and likelihood of seeking medical treatment.

## Conclusion

Our study found that having multiple interventions such as active case isolation and enforcement of safe distancing measures together with improved dormitory design are effective in flattening the epidemic curve. These findings can be used to guide in the planning and design of high-density living areas, while adequately balancing the social interaction and outbreak response needs.

## Methods

Over the course of the pandemic, the Ministry of Health, Singapore (MOH) had over time fine-tuned and adjusted the local COVID-19 suspect case definitions along with the growth and evolution of global evidence base. Under the Infectious Diseases Act in Singapore, suspect COVID-19 cases are required to undergo medical investigation^23^ (Supplementary Table 1) while all pneumonia cases admitted in public hospitals will also be tested for SARS-CoV-2. Furthermore, doctors can conduct discretionary tests on patients based on clinical suspicion or epidemiological risk factors. A confirmed case of acute SARS-CoV-2 infection is defined as a person with respiratory sample positive for SARS-CoV-2 using a laboratory-based reverse transcription polymerase chain reaction (RT-PCR) test^24^.

On March 30, 2020, MOH identified a COVID-19 outbreak in a foreign worker dormitory in Singapore and all epidemiological investigations and outbreak containment measures were implemented under the Infectious Diseases Act Section 59A^23^, which grants the use of outbreak investigation data for analysis and evaluation. As part of the outbreak management, the Ministry is authorised and collected personally identifiable information of the COVID-19 cases. However, for the purpose of the study, only de-identified aggregate numbers were used for analysis and presentation.

### Epidemiological investigation and public health measures

The outbreak response in the dormitory was divided into two phases based on the extent of transmission in the dormitory. In the pre-lockdown phase in the dormitory (before April 6, 2020), all laboratory-confirmed cases were interviewed to collect data on demographic characteristics, clinical symptoms, and activity patterns for the 14 days preceding symptom onset or notification date until isolation in hospital.

Contact tracing was initiated to identify close contacts within the dormitory (persons who stay in the same room as a confirmed case) and in the work and social contexts (persons who spend at least 30 min within 2 m of a confirmed case). These contacts were placed under quarantine for 14 days from last exposure to the case at designated government quarantine facilities located outside the dormitory. The health status of all persons under quarantine was monitored daily and those who developed symptoms would undergo medical investigation as part of active case finding.

To facilitate outbreak investigations and management, details of the dormitory layout and all dormitory residents were requested from the dormitory operator. Employers of a confirmed case and the dormitory operators were also advised to monitor the health condition of all other workers and dormitory residents respectively daily and to advise any person who was unwell to seek medical attention immediately.

The dormitory was locked down on April 6, 2020. During the lockdown phase, healthcare and other workers were deployed to set up medical posts and perform active case finding via swabbing exercises, case isolation and quarantine of close contacts in situ, implementation of safe distancing measures while ensuring the welfare of the dormitory residents. All personnel involved in the dormitory operations were required to observe proper infection control measures at all times and to monitor their health^25^. Residents who were unwell were advised to seek medical attention immediately and were investigated for SARS-CoV-2 infection.

### Sequencing and phylogenetic analysis

All primary samples or residual extracted nucleic acid tested positive for SARS-CoV-2 by RT-PCR at diagnostic laboratories were forwarded to Singapore’s National Public Health Laboratory. Available samples from the dormitory cases and those linked to the dormitory outbreak (e.g. from the workplaces or social interactions) between April 1 and June 6, 2020 were randomly selected for next generation sequencing.

Selected residual diagnostic RNA were subjected to tiled amplicon PCR using ARTIC nCoV-2019 version 3 panel^26^, where One-Step RT-PCR was performed using the SuperScript™ III One-Step RT-PCR System with Platinum™ *Taq* DNA Polymerase (ThermoFisher Scientific). Sequencing libraries were prepared using the Nextera XT and sequenced on MiSeq (Illumina) to generate 300 bp paired-end reads. The reads were subjected to a hard-trim of 50 bp on each side to remove primer artifacts using BBMap^27^ prior to consensus sequence generation by Burrows–Wheeler Aligner-MEM v0.7.17, with default settings. Only sequences with ≥ 98% genome coverage and supported by an average depth of 100 × were included for phylogenetic analysis. The generated consensus sequences were shared via GISAID^28^. To place these sequences into global context, we searched for closely related strains using BLASTN^29^ against all genomes in the GISAID database and retained representative hits with 99.99% identity and matching the time window of our sequences (Acknowledgements in Supplementary Information). The sequences were merged with hCoV-19/Wuhan/WIV04/2019 (accession: EPI_ISL_402124) as reference and root for the tree and aligned using MAFFT (v7.427)^30^. The alignment was manually inspected and trimmed at the 5′ and 3′ ends using Jalview^31^. A maximum likelihood phylogenetic tree was created with IQ-TREE v1.6.1^32^ using ModelFinder^33^ for estimating the best fit model (TN + F in this case) and 1,000 steps of ultrafast bootstrapping^34^ with zero length branches collapsed in the final tree, visualized with Figtree^35^.

### Seroprevalence survey to determine extent of undetected infections

To determine the extent of undetected infections within the dormitory, we undertook a prospective cross-sectional seroprevalence survey in a convenience sample of 7367 dormitory residents who had no travel history 14 days prior to the onset of the first dormitory case and were not previously identified as a confirmed case of acute SARS-CoV-2 infection. Blood samples were collected between May 13 to June 1, 2020 (52–79 days since the onset of the first case; 37–70 days since the lockdown of the dormitory) were tested for SARS-CoV-2 Immunoglobin G (IgG) using either Abbott Architect SARS-CoV-2 IgG assay or Roche Anti-SARS-CoV-2 assay. For the overall seroprevalence, we computed the 95% confidence intervals (CI) for binomial proportions using Wilson’s method^36^.

### Transmission model

Despite the strengthening of public health measures during the lockdown phase, cases continued to rise. This could be attributed to the dense contact networks and living conditions resulting in rapid transmission, the presence of pre- or asymptomatic transmissions, and variable health seeking behaviour of symptomatic persons that could have resulted in a delay or failure to isolate cases who went on to transmit the virus to others. Hence, to estimate the outbreak trajectory within the dormitory and to evaluate the effectiveness of the outbreak control measures, we used an individual-based model of COVID-19 transmission in a simulated population of 12,091 individuals (scaled based on number of individuals in a room) residing in a dormitory with a similar number of blocks, levels and rooms as the dormitory under study (Supplementary Table 2). We assumed that the entire dormitory population was naïve to SARS-CoV-2 infection and disease transmission parameters such as the infectiousness over time and incubation period were modelled based on assumed distributions as elaborated in the Supplementary Information.

### Model fitting

We hypothesized that a diverse range of parameters could drive similar outbreak trajectory in the dormitory. We generated 50,000 random parameter combinations containing parameters related to disease transmission, contact network of the residents within the dormitory, health seeking behaviour or the effectiveness of public health measures (Table 3).

**Table 3:** Range of values for each parameter in a parameter set.

Parameter category	Parameter	Minimum	Maximum
Disease transmission	Initial number of cases	3	20
	Proportion of asymptomatic cases (%)	30	90
	Relative infectiousness of an asymptomatic case (%)	0	50
	Probability of infection inside a room (%)	50	100
	Probability of infection outside a room (%)	0	100
Contact network	Mean number of random contacts form on the same level	0	20
	Mean number of random contacts form on different levels but same block	0	10
	Mean number of random contacts form in different blocks	0	5
Health seeking behaviour	Proportion of symptomatic cases seeking medical attention (%)	0	100
Effectiveness of public health measures	Probability that contacts with persons on the same level remains after social distancing (%)	0	100
	Probability that contacts with persons on different levels of the same block remains after social distancing (%)	0	100
	Probability that contacts with persons in different block remains after social distancing (%)	0	100
	Reduction in probability of infection outside a household (%)	0	50
	Days since deployment of ground officers when reduction in probability of infection outside a household occurred	1	7

The outbreak trajectory in each iteration of a parameter combination was fitted against (i) the number of cases and number of affected locations since the earliest observed onset date (day 1) till day 12 of the outbreak (prior to the lockdown of the dormitory) and (ii) the overall serology outcomes in persons with no recent travel history and no laboratory confirmation of SARS-CoV-2 infection tested by day 79 of the outbreak. For each parameter combination, the full outbreak was simulated ten times to generate 500,000 outputs. The fit of the model against the observed data in the early and late phases of the outbreak was determined by computing the likelihood (Supplementary Information). Observed case counts in the lockdown phase were not used for model fitting as symptom onset dates were not routinely collected.

### Outbreak interventions scenarios

Parameter combinations were assigned a weight based on the corresponding likelihood and weighted sampling of the parameter combinations with replacement was performed 10,000 times. We simulated the current outbreak scenario with interventions including the deployment of ground teams to expedite case isolation, quarantine of roommates, enforcement of physical distancing and reduction in probability of infection outside the room in the lockdown phase of the outbreak (Supplementary Information). Using the same disease transmission and contact network parameters, we also simulated alternative scenarios of the dormitory outbreak: (i) baseline scenario: only case isolation and quarantine of roommates were implemented; (ii) enhanced response and physical distancing scenario: ground teams were deployed to expedite case isolation and enforce physical distancing measures but no strengthening of measures was applied to reduce probability of infections occurring outside a room in the lockdown phase of the outbreak; (iii) modified dormitory setting scenario: dormitory layout with reduced number of residents per room and, en suite bathroom, shower and cooking facilities to ensure that persons under quarantine did not leave their rooms and movement restrictions across different levels for the remaining residents when the dormitory was under lockdown^10^. Details of the respective interventions in each scenario are described in the Supplementary Information.

#### Outbreak metrics

For each parameter combination, disease progression was tracked over time and generations (${g}_{x}$ where the subscript $x$ indicates the respective generation). The reproduction number of the $x$th generation—i.e. the ratio of cases in consecutive generations $\left(\text{i.e. }\frac{{g}_{x+1}}{{g}_{x}}\right)$ which provides an indication of the growth of an outbreak—was determined. Furthermore, we estimated the final attack rate, the duration of the full outbreak, the peak outbreak size and time to outbreak peak in all scenarios. We performed a Welch’s t-test to evaluate the outbreak metrics of each alternative scenario against the current outbreak scenario and p values < 0.05 were considered statistically significant. All analyses were done using R version 3.5.1^37^. A full description of the model is available in the Supplementary Information.

A full record of the data is maintained at the Ministry of Health, Singapore and samples that were selected for next generation sequencing are uploaded to GSAID. These details can be made available through formal request.

## Supplementary Information

Supplementary Information.
