# Supplementary material for: Factors influencing SARS-CoV-2 transmission and outbreak control measures in densely populated settings
Source: Sci Rep. 2021 Jul 27;11:15297. doi: 10.1038/s41598-021-94463-3 (PMC8316572; doi:10.1038/s41598-021-94463-3)
Supplement: Supplementary file 1 — Supplementary Information. [file 41598_2021_94463_MOESM1_ESM.docx]

**Factors influencing SARS-CoV-2 transmission and outbreak control measures in densely populated settings - Supplementary Info**

Rachael Pung*^1^, Bernard Lin*^3^, Sebastian Maurer-Stroh^4,5,6^, Fernanda L Sirota^4^, Tze Minn Mak^6^, Sophie Octavia^6^, Junxiong Pang^1,7,8^, Iain Beehuat Tan^1,9,10,11^, Clive Tan^7,12^, Biauw Chi Ong^11,13,14^, Alex R Cook^7,8^, Vernon J Lee^1,7^ on behalf of the Singapore COVID-19 Outbreak Research team

*These authors contributed equally to the paper

^1^ Ministry of Health, Singapore

^2^ Ministry of Manpower, Singapore

^3^ Government Technology Agency, Singapore

^4^ Bioinformatics Institute, Agency for Science, Technology and Research, Singapore

^5^ Department of Biological Sciences, National University of Singapore, Singapore

^6^ National Public Health Laboratory, National Centre for Infectious Diseases, Singapore

^7^ Saw Swee Hock School of Public Health, National University of Singapore

^8^ National University Health System, Singapore

^9^ Department of Medical Oncology, National Cancer Centre, Singhealth

^10^ Genome Institute of Singapore, Agency for Science, Technology and Research, Singapore

^11^ Duke-NUS Medical School

^12^ Singapore Armed Forces Headquarters Medical Corps

^13^ Sengkang General Hospital, Singhealth

^14^ Singhealth Duke-NUS Academic Medical Centre

**Supplementary Table 1:** Suspect case definitions used in Singapore.

| **Date** | **Suspect case definition** |
| --- | --- |
| 2 January 2020 (Initial) | 1. A person with clinical signs and symptoms suggestive of pneumonia or severe respiratory infection with breathlessness **AND** travel to or residence in **Wuhan city** within the last 14 days; or 2. A person with an acute respiratory illness of any degree of severity who, within 14 days before onset of illness, had **close contact^1^** with a pneumonia case of unknown cause linked to the Wuhan cluster. |
| 21 January 2020 | 1. A person with clinical signs and symptoms suggestive of pneumonia or severe respiratory infection with breathlessness AND travel to **mainland China** within 14 days before onset of illness; or 2. A person with an acute respiratory illness of any degree of severity who, within 14 days before onset of illness, had **been to a hospital in mainland China** or had close contact^1^ with a case of 2019 novel coronavirus infection. |
| 25 January 2020 | 1. A person with clinical signs and symptoms suggestive of pneumonia or severe respiratory infection with breathlessness AND travel to mainland China within 14 days before onset of illness; or 2. A person with an acute respiratory illness of any degree of severity who, within 14 days before onset of illness had: 3. Been to **Wuhan city or Hubei Province, China**; OR 4. Been to a hospital in mainland China; OR 5. Had close contact^1^ with a case of 2019 novel coronavirus infection. |
| 4 February 2020 | 1. A person with clinical signs and symptoms suggestive of pneumonia or severe respiratory infection with breathlessness AND travel to mainland China within 14 days before onset of illness; or 2. A person with an acute respiratory illness of any degree of severity who, within 14 days before onset of illness had: 3. Been to **Hubei Province (including Wuhan city) or Zhejiang Province (including Hangzhou city)**, China; OR 4. Been to a hospital in mainland China; OR 5. Had close contact^1^ with a case of 2019 novel coronavirus infection; OR 6. Had **frequent or close contact during work^2^** **with recent travellers from mainland China** (travel history in the last 14 days). |
| 9 March 2020 | 1. A person with clinical signs and symptoms suggestive of pneumonia or severe respiratory infection with breathlessness AND who within 14 days before onset of illness had travelled abroad (i.e. to any country outside of Singapore). 2. A person with an acute respiratory illness of any degree of severity who, within 14 days before onset of illness had: 3. Been to any of the **areas requiring heightened vigilance**^3^ as listed on the Healthcare Professionals Portal: https://www.moh.gov.sg/hpp/all-healthcare-professionals; OR 4. Been to **any hospital abroad**; **OR** 5. Close contact^4^ with a case of COVID-19 infection. |
| 16 April 2020 | 1. A person with clinical signs and symptoms suggestive of Community-Acquired Pneumonia^5^ or ­community-acquired severe respiratory infection with breathlessness. 2. A person with an acute respiratory illness of any degree of severity (e.g. symptoms of cough, sore throat, runny nose, anosmia), with or without fever, who, within 14 days before onset of illness had: 3. Travelled abroad (outside Singapore); OR 4. Close contact^4^ with a case of COVID-19 infection OR 5. Stayed in a foreign worker dormitory^6^ |
| 8 May 2020 | 1. A person with clinical signs and symptoms suggestive of Community-Acquired Pneumonia^5^ 2. A person with an acute respiratory illness of any degree of severity (e.g. symptoms of cough, sore throat, runny nose, anosmia), with or without fever, who, within 14 days before onset of illness had: 3. Travelled abroad (outside Singapore); OR 4. Close contact^4^ with a case of COVID-19 infection OR 5. Stayed in a foreign worker dormitory^6^ 6. Worked in occupations or environments with higher risk of exposure to COVID-19 cases^7^ 7. Any person with prolonged febrile^8^ acute respiratory infection (ARI) symptoms of 4 days or more, and not recovering |
| 1. June 2020 | 1. A person with clinical signs and symptoms suggestive of Community-Acquired Pneumonia^5^ 2. A person with an acute respiratory illness of any degree of severity (e.g. symptoms of cough, sore throat, runny nose, anosmia), with or without fever, who, within 14 days before onset of illness had: 3. Travelled abroad (outside Singapore); OR 4. Close contact^4^ with a case of COVID-19 infection OR 5. Stayed in a foreign worker dormitory^6^ 6. Worked in occupations or environments with higher risk of exposure to COVID-19 cases^7^ 7. Any person with prolonged febrile^8^ acute respiratory infection (PARI) symptoms of 4 days or more, and not recovering AND who had not undergone prior swabbing for ARI symptoms in the same episode of illness. |

^1^ Close contact is defined as: Anyone who provided care for the patient, including a health care worker or family member, or who had other similarly close physical contact; Anyone who stayed (e.g. lived with, visited) at the same place as a case.

^2^ Persons who attended business meetings/discussions, frontline staff in hospitality and tourism (e.g. hotels, shops, tours) with regular dealings with travellers from mainland China.

^3^ Refers to affected areas with high disease load or high connectivity to Singapore. As of 9 March 2020, these are: Mainland China, Republic of Korea, Italy, Iran, France, Germany, Spain, Japan and the United Kingdom. Please check the Healthcare Professionals Portal regularly for updates.

^4^ Close contact is defined as: Anyone who provided care for the patient, including a health care worker or family member, or who had other similarly close physical contact; Anyone who stayed (e.g. household members) at the same place as a case; Anyone who had close (i.e. less than 2m) and prolonged contact (30 min or more) with a case (e.g. shared a meal).

^5^ Excludes cases of nosocomial pneumonia and aspiration pneumonia with no links to confirmed cases

^6^ Separate processes apply to foreign workers from a dormitory that has dedicated medical station /clinic or dedicated workflow for assessment and swabbing

^7^ These include but are not limited to any staff (healthcare worker and non-healthcare worker) working in:

- Public and private healthcare settings, spanning acute care, primary care, intermediate and long-term care and community care settings
- Dormitories or involved in dormitory outbreak control operations
- Isolation / quarantine facilities
- Community care facilities (CCFs)/ community recovery facilities (CRFs)
- Ambulance and dedicated patient transport (including private hire vehicles).

^8^ Fever, of any duration, with measured or reported temperature of > 37.5ºC.

**Supplementary Table 2: Layout of dormitory**

| **Variables** | **Dormitory characteristics** |
| --- | --- |
| No. of occupants | 12 000 – 13 000 |
| Layout |  |
| No. of zones with restricted access | 3 |
| No. of blocks per zone | 4–5 |
| No. of levels per block | 4 |
| No. of rooms per level | 25 |
| No. of individuals per rooms | 10–12 |
| Facilities |  |
| Toilet/Shower | Shared facility on each level |
| Cooking/Dining area | Ground level of each block |
| Recreational spaces | Accessible to all occupants |

# **Transmission model**

## Dormitory contact network parameters

We simulated a dormitory comprising 11 blocks, with 4 levels per block and 25 rooms per level. The number of individuals per room was modelled using a uniform distribution ranging from 10 to 12 persons. The resulting dormitory population size was 12 091 individuals, which reflects the capacity of the dormitory under study.

Given the close living conditions in a room, we assumed all persons in the room have contact with each other. Each individual had an average of 10 close contacts in a room (i.e. the mean number of individuals per room minus the individual in question).

Outside a room, the number of random contacts formed on the same level, same block, or other parts of the dormitory was assumed to follow a Poisson distribution and the mean number of random contacts for each location was modelled according to a range of values listed in Table 1 main text. The mean number of random contacts formed by each individual for each location is analogous to the mean degree of a vertex in a contact network. These contacts remains unchanged (i.e. static network) unless physical distancing or quarantine is implemented.

## Disease transmission parameters

We assumed that the incubation period follows a gamma distribution (shape = 5.807, scale = 0.948; i.e. $\mu$ = 5.505, $\sigma$ = 2.2845)^1,2^ and symptomatic individuals are infectious 3 days before symptoms onset and up to 14 days after symptoms onset.^3–5^

Based on Singapore’s case data, both published^6^ and unpublished, the Gamma distribution provided for a better fit of the data. The estimated lognormal distribution in Lauer et al. has a heavier tail while the Weibull distribution has a higher probability for incubation periods less than two days and hence these parameters were not selected for modelling. However, we noted that all the derived parameter estimates for various parametric distributions of the incubation period of SARS-CoV-2 using 181 confirmed cases in Lauer et al, were similar to the base model used in their analysis (i.e. log normal model). As such, these differences in the assumed distributions would have minimal impact to the overall transmission process. Recent review by Quesada et al. also yielded similar parameter estimates to our assumed Gamma distributed incubation period.

We assumed that exposure to susceptible individuals follows a gamma distribution (shape = 2.5, rate = 0.5; i.e. $\mu$= 5, $\sigma$ = 3.1623) since the start of the infectious period of a case to allow for pre-symptomatic infections to occur approximately 30% of the time (within the estimated range presented by Ferretti et al. (2020))^7^. Pre-symptomatic cases are equally infectious as symptomatic cases.

Asymptomatic cases are also able to transmit infections with a lowered level of infectiousness. We assign a pseudo time of symptom onset for these cases following the same gamma distributed incubation period for symptomatic cases in order to define their infectious period same as that of symptomatic cases.

The proportion of asymptomatic cases, the relative infectiousness of an asymptomatic case, the probability of infection inside a room and the probability of infection outside a room was modelled according to a range of values listed in Table 1 main text. Both probabilities of infection applies to the entire duration of contact between a case and a susceptible individual.

## Outbreak intervention parameters

(i) Case isolation

For symptomatic cases that sought medical attention, case isolation was assumed to occur at most 3 days (uniform distribution min = 0 days, max = 3 days) after the onset of symptoms and this is reduced to at most 1 day after the deployment of ground officers 14 days since the earliest observed onset date (i.e. day 15).

The probability of a symptomatic case seeking medical attention was modelled according to a range of values listed in Table 1 main text.

(ii) Quarantine of close contacts

Throughout the simulation, contacts in the same room as a confirmed case would be quarantined in situ with other roommates. Persons under quarantine would spend most of their time confined to their room but would continue to share shower and toilet facilities with others on the same level only. This translates to lowered number of contacts formed with others on the same level but cessation of contacts with others in other parts of the dorm.

Under a modified dormitory setting, en suite facilities help to ensure that persons under quarantine do not leave their rooms (i.e. number of contacts formed with outside the room for persons under quarantine is reduced to zero)

(iii) Social distancing

Ground officers were deployed to implement social distancing measures in the dormitory 14 days since the earliest observed onset date (i.e. day 15). The reduction in the mean number of random contacts for each location was modelled according to a range of values listed in listed in Table 1 main text.

Under a modified dormitory setting scenario, en suite facilities help to ensure that no mixing of residents who are not under quarantine across different levels and different parts of the dormitory when the dormitory is placed under lockdown (i.e. For persons not under quarantine, the number of contacts formed with others on different levels and in different blocks is reduced to zero and there is a reduction in mean number of contacts formed on the same level).

(iv) Reduction in probability of infection outside the household

In the actual outbreak situation, majority of the residents have restricted movement in the dormitory, heighten awareness of the situation and practice mask wearing since the deployment of ground officers. Hence, the probability of infection upon contact with a case outside the room is assumed to decrease.

The reduction in the probability of infection outside the household and the time which this reduction occurred was modelled according to a range of values listed in Table 1 main text.

We hypothesized that a diverse range of parameters could drive similar outbreak trajectory in dormitory under study. We generated 50,000 random parameter combinations with parameters in Table 1 main text.

# **Model Fitting**

We denote $c(t)$ as the number of observed cases with symptom onset on day $t$ (with day 1 as the day of the earliest symptom onset in the observed case(s)) and $R\left( t \right), L\left( t \right), B(t)$ as the cumulative number of rooms, levels and blocks in a dormitory with observed cases by day $t$. $\theta$ is the combination of parameters in Table 1 main text for the current iteration.

We assume that $c(t)$ is drawn from a Poisson distribution with mean $\lambda_{c}(t)$, the number of modelled cases with symptom onset on day $t$ where

$$\lambda_{c}\left( t \right)=M(\lambda_{c}\left( t-1 \right), \theta)$$

and the modelled cases with the earliest onset on $t=1$ is dependent on the initial number of cases, the probability of a case being symptomatic, probability of a symptomatic case seeking medical attention (i.e. parameters in $\theta$), and the model specification to give the following:

$$\lambda_{c}\left( 1 \right)=M(\theta)$$

We also assume that $R\left( t \right), L\left( t \right), B(t)$ are each drawn from a Poisson distribution with mean $\lambda_{R}\left( t \right), \lambda_{L}\left( t \right), \lambda_{B}(t)$ in the respective order, the cumulative number of rooms, levels and blocks in a dormitory with modelled cases by day $t$. The formulation of $\lambda_{R}\left( t \right), \lambda_{L}\left( t \right), \lambda_{B}(t)$ follows the formulation of $\lambda_{c}\left( t \right)$.

We denote $S$ as the number of serology positive residents by day 79 of the outbreak among $N$ eligible dormitory residents who participated in the seroprevalence survey. We assumed the observed seroprevalence follows a Binomial distribution parameterized by $s$, the modelled seroprevalence by day 79.

For an iteration, $i$, and a given parameter combination, $p$, we define the likelihood of observing the cases, affected rooms, levels, units up to day 12 and the seroprevalence on day 79 as:

$$L_{i,p}= \prod_{t=1}^{12} P_{pois}\left[ c\left( t \right) | \lambda_{c,i,p}(t) \right] P_{pois}\left[ R\left( t \right) | \lambda_{R,i,p}\left( t \right) \right] P_{pois}\left[ L\left( t \right) | \lambda_{L,i,p}\left( t \right) \right] P_{pois}\left[ B(t) | \lambda_{B,i,p}(t) \right]$$

$$\times P_{binom}\left[ S,N | s_{i,p} \right]$$

The first line gives the probability of the number of cases, affected rooms, levels and blocks up to day 12 of the outbreak (changes in data collection method occurred after day 12 and hence subsequent data not used for model validation). The second line gives the probability of the seroprevalence on day 79 of the outbreak.

# **Modelling outbreak intervention scenarios**

Each iteration for a parameter combination is assigned a weight as follows:

$$w_{i,p}= \frac{L_{i,p}}{\sum_{i} \sum_{p} L_{i,p}}$$

and we resampled the parameter combinations proportional to $w_{i,p}$. Resampling of the parameter combination with replacement is performed for 10,000 times before simulating all 4 outbreak interventions scenario with the resampled parameter combinations (Supplementary Table 3).

The respective outbreak scenarios and the corresponding public health measures are listed in Supplementary Table 4. For scenarios where social distancing and/or reduction in probability of infection outside the room is not implemented during the lockdown phase, the parameters under the category “Effectiveness of public health measures” in Supplementary Table 3 will not be applicable.

# **Supplementary Table 3: Unique sampled parameter combinations**

| Parameter category | Parameter set | 1 | 2 | 3 | 4 | 5 | 6 | 7 | 8 | 9 | 10 |
| --- | --- | --- | --- | --- | --- | --- | --- | --- | --- | --- | --- |
| Disease transmission | Initial number of cases | 13 | 17 | 14 | 15 | 10 | 17 | 14 | 8 | 13 | 16 |
|  | Proportion of asymptomatic cases (%) | 88.2 | 40.1 | 75.3 | 87.9 | 88.5 | 65.6 | 84.6 | 80.4 | 81.4 | 66.3 |
|  | Relative infectiousness of an asymptomatic case (%) | 11.0 | 35.4 | 12.9 | 10.8 | 38.2 | 46.5 | 7.9 | 3.6 | 24.2 | 13.3 |
|  | Probability of infection inside a room (%) | 50 | 55 | 75 | 91 | 79 | 73 | 76 | 83 | 54 | 82 |
|  | Probability of infection outside a room (%) | 90 | 16 | 84 | 76 | 34 | 17 | 84 | 92 | 71 | 45 |
| Contact network | Mean number of random contacts form on the same level | 16.2 | 12.0 | 8.6 | 13.8 | 12.9 | 15.2 | 14.3 | 6.3 | 3.3 | 7.4 |
|  | Mean number of random contacts form on different levels but same block | 3.6 | 5.3 | 2.1 | 4.7 | 10.0 | 7.6 | 8.8 | 6.1 | 4.8 | 3.6 |
|  | Mean number of random contacts form in different blocks | 3.7 | 0.3 | 3.2 | 3.5 | 0.2 | 0.6 | 3.0 | 4.5 | 2.6 | 2.2 |
| Health seeking behaviour | Proportion of symptomatic cases seeking medical attention (%) | 87.6 | 10.0 | 61.2 | 86.4 | 54.2 | 18.4 | 36.7 | 36.5 | 62.8 | 37.8 |
| Effectiveness of public health measures | Probability that contacts with persons on the same level remains after social distancing (%) | 55 | 2 | 3 | 49 | 48 | 43 | 32 | 14 | 50 | 23 |
|  | Probability that contacts with persons on different levels of the same block remains after social distancing (%) | 57 | 46 | 10 | 3 | 46 | 63 | 6 | 38 | 2 | 26 |
|  | Probability that contacts with persons in different block remains after social distancing (%) | 72 | 93 | 90 | 45 | 87 | 3 | 9 | 25 | 36 | 56 |
|  | Reduction in probability of infection outside a household (%) | 32 | 22 | 32 | 30 | 5 | 1 | 22 | 33 | 37 | 24 |
|  | Days since deployment of ground officers when reduction in probability of infection outside a household occurred | 2.2 | 6.3 | 2.1 | 4.0 | 5.1 | 3.7 | 3.8 | 6.4 | 1.9 | 2.3 |

| Parameter category | Parameter set | 11 | 12 | 13 | 14 | 15 | 16 | 17 | 18 | 19 | 20 |
| --- | --- | --- | --- | --- | --- | --- | --- | --- | --- | --- | --- |
| Disease transmission | Initial number of cases | 9 | 19 | 12 | 15 | 16 | 6 | 14 | 19 | 18 | 12 |
|  | Proportion of asymptomatic cases (%) | 58.4 | 77.4 | 69.6 | 67.9 | 51.7 | 73.1 | 66.0 | 86.2 | 50.0 | 55.9 |
|  | Relative infectiousness of an asymptomatic case (%) | 25.4 | 13.0 | 16.4 | 10.6 | 0.9 | 44.5 | 3.5 | 23.4 | 8.7 | 0.2 |
|  | Probability of infection inside a room (%) | 82 | 84 | 50 | 54 | 64 | 55 | 81 | 51 | 91 | 72 |
|  | Probability of infection outside a room (%) | 20 | 69 | 46 | 72 | 20 | 99 | 24 | 73 | 14 | 50 |
| Contact network | Mean number of random contacts form on the same level | 6.7 | 8.2 | 12.6 | 0.3 | 12.5 | 2.2 | 16.1 | 11.5 | 10.3 | 8.1 |
|  | Mean number of random contacts form on different levels but same block | 3.6 | 4.3 | 3.0 | 7.1 | 5.2 | 0.7 | 8.8 | 1.7 | 4.8 | 3.9 |
|  | Mean number of random contacts form in different blocks | 4.0 | 3.8 | 2.8 | 3.0 | 4.7 | 0.8 | 4.8 | 0.1 | 2.4 | 2.0 |
| Health seeking behaviour | Proportion of symptomatic cases seeking medical attention (%) | 13.5 | 46.8 | 77.7 | 37.1 | 21.7 | 68.0 | 25.0 | 53.1 | 15.6 | 18.7 |
| Effectiveness of public health measures | Probability that contacts with persons on the same level remains after social distancing (%) | 23 | 19 | 43 | 29 | 50 | 87 | 66 | 15 | 49 | 70 |
|  | Probability that contacts with persons on different levels of the same block remains after social distancing (%) | 18 | 54 | 50 | 60 | 5 | 44 | 95 | 48 | 95 | 80 |
|  | Probability that contacts with persons in different block remains after social distancing (%) | 35 | 76 | 49 | 99 | 18 | 48 | 70 | 43 | 25 | 32 |
|  | Reduction in probability of infection outside a household (%) | 13 | 04 | 44 | 14 | 3 | 3 | 9 | 44 | 27 | 2 |
|  | Days since deployment of ground officers when reduction in probability of infection outside a household occurred | 1.3 | 3.0 | 6.5 | 3.4 | 6.4 | 4.0 | 3.0 | 6.3 | 1.6 | 4.0 |

**Supplementary Table 3 Outbreak intervention scenarios**

| **Scenarios** | **Public health measures** |
| --- | --- |
| Current outbreak scenario | - Deployment of ground teams to expedite case isolation - Quarantine of roommates - Enforcement of physical distancing - Reduction in probability of infection outside the room in the lockdown phase of the outbreak |
| Baseline scenario | - Case isolation - Quarantine of roommates |
| Enhanced response and physical distancing scenario | - Deployment of ground teams to expedite case isolation - Quarantine of roommates - Enforcement physical distancing measures |
| Modified dormitory setting scenario | - Deployment of ground teams to expedite case isolation - Quarantine of roommates - Enforcement of physical distancing - Reduction in probability of infection outside the room in the lockdown phase of the outbreak - Reduced number of residents per room and, en suite bathroom, shower and cooking facilities to ensure that persons under quarantine did not leave their rooms - Movement restrictions across different levels for the remaining residents when the dormitory was under lockdown |

**References**

1 Lauer SA, Grantz KH, Bi Q, *et al.* The Incubation Period of Coronavirus Disease 2019 (COVID-19) From Publicly Reported Confirmed Cases: Estimation and Application. *Ann Intern Med* 2020; **172**: 577–82.

2 Quesada JA, López-Pineda A, Gil-Guillén VF, Arriero-Marín JM, Gutiérrez F, Carratala-Munuera C. Incubation period of COVID-19: A systematic review and meta-analysis. *Rev Clin Esp (Barc)* 2021; **221**: 109–17.

3 Wei WE. Presymptomatic Transmission of SARS-CoV-2 — Singapore, January 23–March 16, 2020. *MMWR Morb Mortal Wkly Rep* 2020; **69**. DOI:10.15585/mmwr.mm6914e1.

4 Furukawa NW, Brooks JT, Sobel J. Evidence Supporting Transmission of Severe Acute Respiratory Syndrome Coronavirus 2 While Presymptomatic or Asymptomatic - Volume 26, Number 7—July 2020 - Emerging Infectious Diseases journal - CDC. DOI:10.3201/eid2607.201595.

5 Byrne AW, McEvoy D, Collins AB, *et al.* Inferred duration of infectious period of SARS-CoV-2: rapid scoping review and analysis of available evidence for asymptomatic and symptomatic COVID-19 cases. *BMJ Open* 2020; **10**: e039856.

6 Pung R, Chiew CJ, Young BE, *et al.* Investigation of three clusters of COVID-19 in Singapore: implications for surveillance and response measures - The Lancet. *Lancet* 2020; **395**: 1039–46.

7 Ferretti L, Wymant C, Kendall M, *et al.* Quantifying SARS-CoV-2 transmission suggests epidemic control with digital contact tracing. *Science* 2020; **368**: eabb6936.
